# A Rare Cause of Calcified Subdural Empyema and Ventriculitis in a Pediatric Patient: *Achromobacter Denitrificans*

**DOI:** 10.5334/jbr-btr.925

**Published:** 2016-02-02

**Authors:** Mehtap Beker-Acay, Mehmet Gazi Boyaci, Gulsah Asik, Resit Koken, Ebru Unlu, Usame Rakip

**Affiliations:** 1 Department of Radiology, Afyon Kocatepe University, Faculty of Medicine, Afyonkarahisar, Turkey; 2 Department of Neurosurgery, Afyon Kocatepe University, Faculty of Medicine, Afyonkarahisar, Turkey; 3 Department of Microbiology, Afyon Kocatepe University, Faculty of Medicine, Afyonkarahisar, Turkey; 4 Department of Pediatrics, Afyon Kocatepe University, Faculty of Medicine, Afyonkarahisar, Turkey

**Keywords:** Achromobacter denitrificans, Subdural empyema, Ventriculitis

## Abstract

Intracranial infections in the pediatric age group are still important causes of morbidity in developing countries. A 2-year-old male patient presented with acute onset of seizures and loss of consciousness to our emergency department with a past history of being followed for hypogammaglobulinemia. Unenhanced computerized tomography scan of the brain revealed a right frontoparietal peripherally calcified extraaxial collection, brain edema and a left sided shift. Contrast enhanced magnetic resonance imaging revealed a subdural empyema associated with the brain parenchyma and the ventricular system. In spite of a decompression procedure and subsequent medical therapy, the patient succumbed on the 9. postoperative day. This is the first case report of a pediatric patient with subdural empyema and ventriculitis due to Achromobacter denitrificans.

## Introduction

Intracranial infections in the pediatric age group are still important causes of morbidity despite the advances in imaging techniques, bacterial isolation technology and newly developed broad-spectrum antibiotics, particularly in developing countries [1]. Achromobacter denitrificans, also called as Alcaligenes denitrificans is a subspecies of genus achromobacter [2,3,4]. The most important member of this family that was evaluated strictly in the literature is Achromobacter xylosoxidans. It is an opportunistic agent of various, predominantly nasocomial infections usually in immunocompromised patients [2]. It was isolated from soil and aquatic environments, also from the normal flora of the ear and gastrointestinal tract in humans [2,3]. It can cause symptomatic infections ranging from natural or prosthetic valve endocarditis to meningitis, pneumonia, peritonitis, conjunctivitis, osteomyelitis, intra-abdominal abscess, and prosthesis infections [4]. Here we present a case report of a pediatric patient with subdural empyema and ventriculitis due to Achromobacter denitrificans which was not described in the medical literature before.

## Case Report

**Figure 1:**
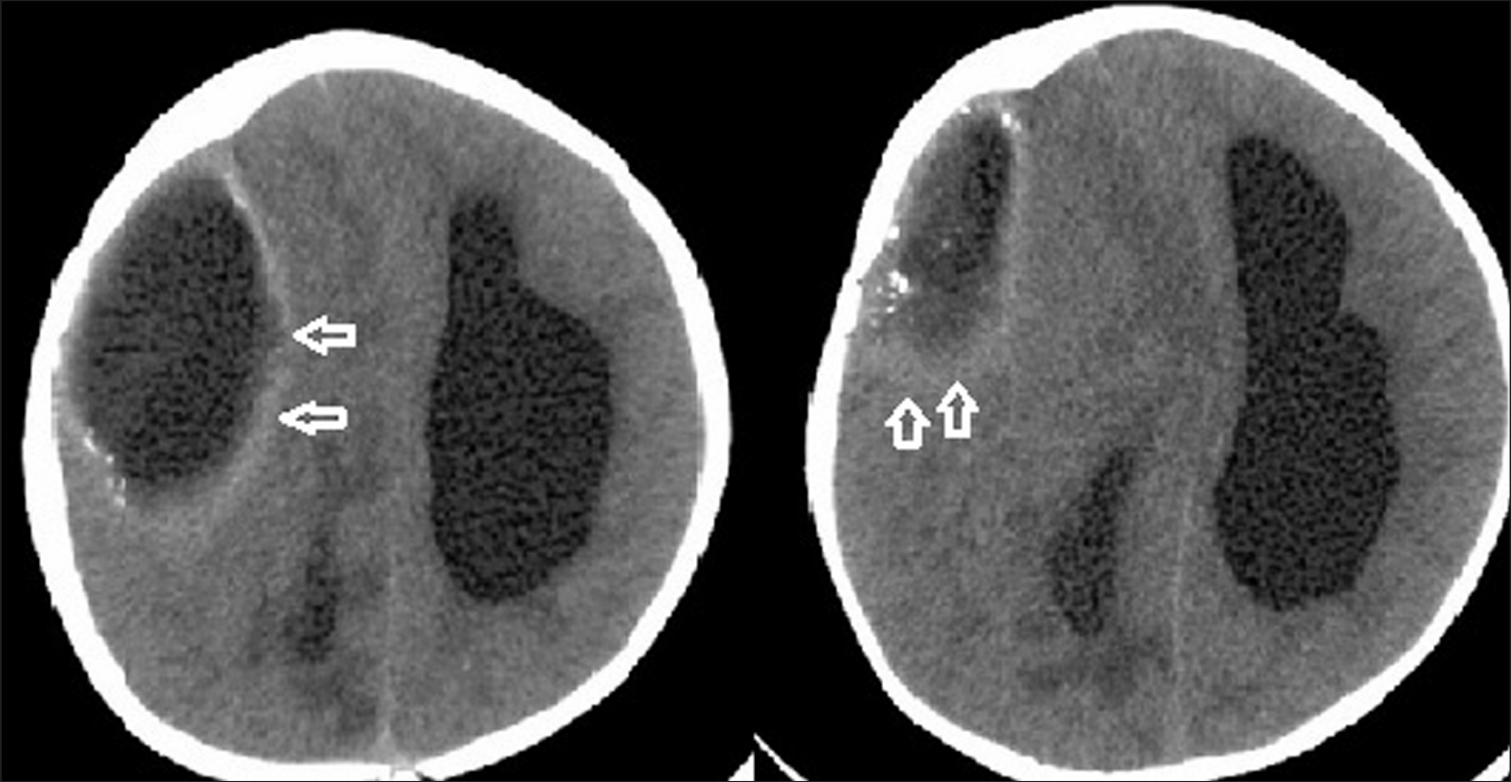
Noncontrast CT images detected a 4 cm right frontoparietal extraaxial collection with thick, calcified walls (arrows). Also, brain edema, hydrocephaly and subfalcine herniation were observed.

**Figure 2:**
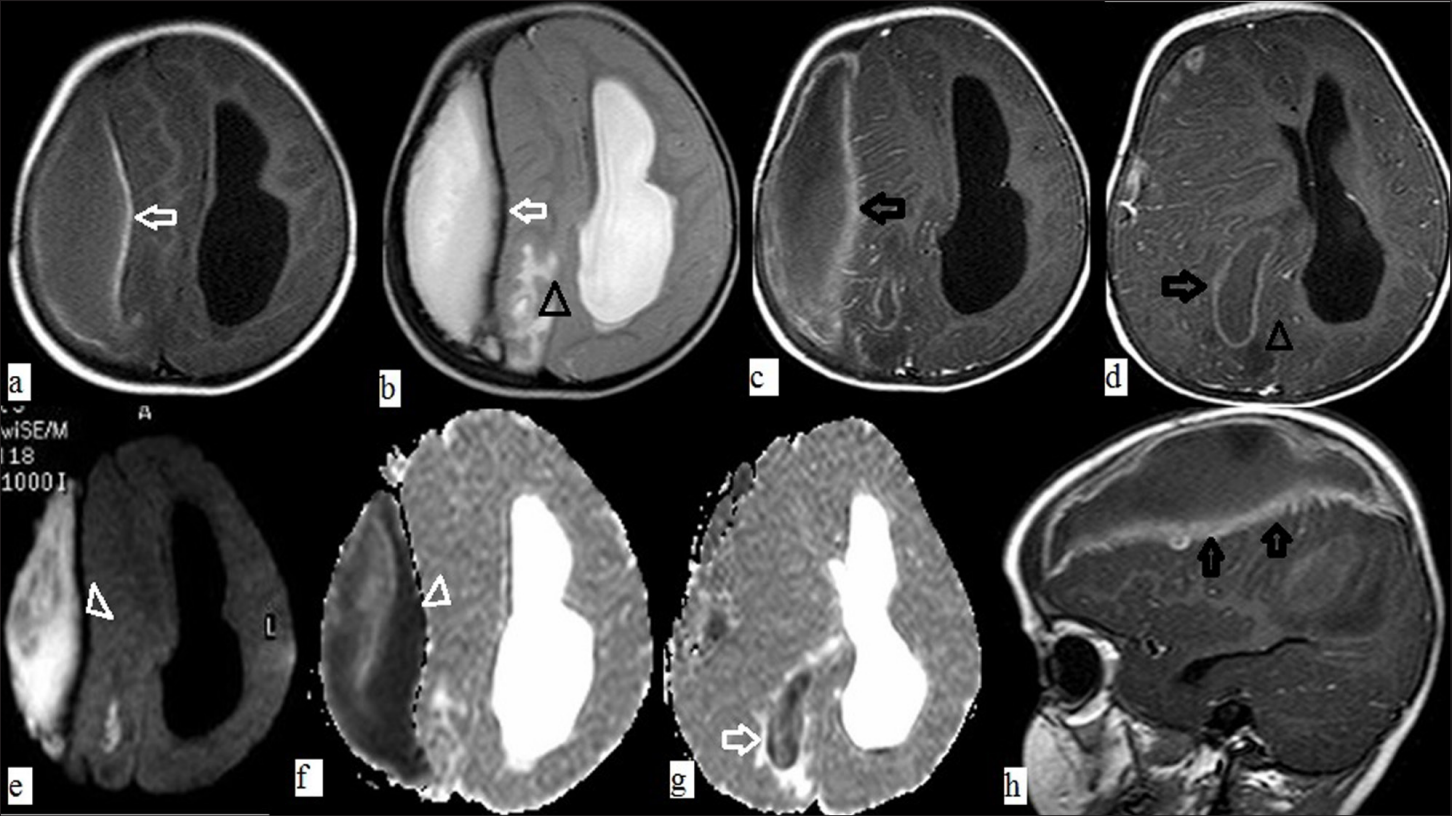
On MR imaging, walls of the subdural collection are hyperintense on noncontrast axial T1- (a, white arrow) and hypointense on axial T2-weighted (b, white arrow) images compatible with calcification. On isotropic b 1000 image (e) and ADC maps (f, g) restricted diffusion in the infected areas (white arrowheads) as well as in the occipital horn of the lateral ventricle (image g, white arrow), consistent with pus accumulation were observed. On contrast enhanced axial (c, d) and sagittal T1-weighted (h) images, abscess wall was irregularly enhancing and increased ependymal enhancement within the lateral ventricles as well as of the overlying meninges are observed (black arrows). Finally, intraparenchymal and intraventricular extension with surrounding vasogenic edema can be seen (black arrowheads).

**Figure 3:**
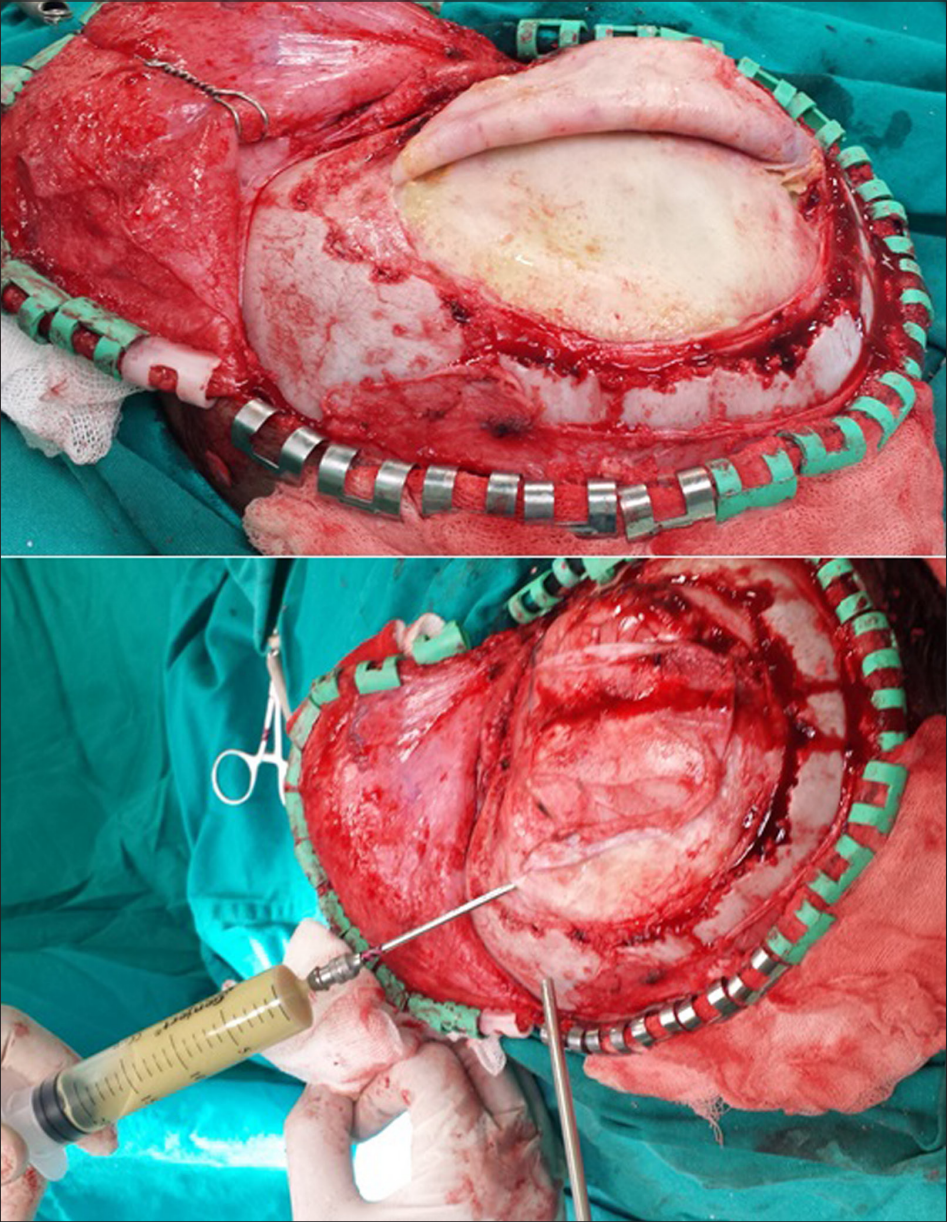
Intraoperative photograph showing a fibrotic and thickened abscess wall. Purulent material was aspirated and abscess walls were excised.

A 2-year-old male patient presented with acute onset of seizures and loss of consciousness to our emergency department. His past history revealed that he was being followed for hypogammaglobulinemia over the past year without any change of consciousness nor seizures. He had no history of head injury, trauma, or infectious diseases. Neurological examination disclosed a Glasgow Coma Score (GCS) of 3, his pupillary were anisocoric, direct light reflex and indirect light reflex were found to be negative. The laboratory examinations showed: ESR 120, CRP 6.27 mg/dL, white blood cell count 18,400 μL (neutrophils 70%) and platelets 512,000/μL. Unenhanced computerized tomography scan of the brain revealed a right frontoparietal peripherally calcified extraaxial collection, brain edema and a left sided shift (Fig. 1). A contrast enhanced magnetic resonance imaging showed the presence of a subdural empyema associated with the ipsilateral lesion in the brain parenchyma and extension of the infection to the ventricular system was demonstrated (Fig. 2). Neurological situation showed brain herniation symptoms, so the patient underwent emergency operation. A right sided wide decompressive craniectomy with extensive trauma incision was performed. Dura mater was tense and fibrotic, when it was passed a 15 × 7 cm fibrotic and thickened abscess with full of pus was encountered. An aspiration about 50 cc of purulent material was discharged and abscess walls were excised (Fig. 3). A decompression procedure was performed for the parenchymal mass and 15 cc collection was aspirated. Postoperative imaging studies disclosed the successful decompression procedure. Aspirate samples showed numerous polymorphonuclear leukocytes without bacteria at gram staining, cultures were positive for Achromobacter denitrificans, negative for anaerobic bacteria or fungi. The isolate was susceptible to ampicillin-sulbactam, trimethoprim-sulfamethoxazol, tigecycline, ceftazidime, levofloxacin and ciprofloxacin. But in spite of subsequent antibiotic therapy patient’s neurological situation got worse in the postoperative period. The patient’s GCS remained 3 and he succumbed on the postoperative day 9.

## Discussion

Achromobacter denitrificans is a motile, oxidase-positive, catalase-positive, aerobic bacterium of genus achromobacter not fermenting glucose [3,4]. It has been isolated from soil and aquatic environments, also in the endogenous flora [3,5,6]. Achromobacter xylosoxidans, another sub-species of genus achromobacter is the causative agent of mostly nosocomial infections isolated from human samples. The sources of infection may be contaminated fluids, haemodialysis fluid, mouth irrigation fluid, incubators, nebulizers, soap, and disinfectants in the hospitals [6]. Our patient had no history of penetrating head trauma nor a catheterization. He might have had this causative agent from the enviromental sources. This pathogen can cause natural/prosthetic valve endocarditis, meningitis, pneumonia, conjunctivitis, osteomyelitis, peritonitis, urinary tract infections, surgical area infections, sepsis and intra-abdominal abscess [3,6,7].

The mortality rate of Achromobacter xylosoxidans infections ranging from 3% to 80% depends on the patients’ age, immune competancy and whether it is hospital- or community-acquired, and also on the type of infection [2,6]. Mortality rates among neonates with meningitis are generally high [8]. The identified risk factors for Achromobacter infections are immunodeficiency, HIV infection, malignancy, cystic fibrosis, prematurity, hypoglobulinemia and hospitalization [5]. Our patient had hypogammaglobulinemia which may contribute to reduced ability of effective antibody responses. Although high levels of resistance to cephalosporin, aminoglycoside, and quinolone have been reported, it is usually sensitive to common antibiotics like cotrimoxazole, piperacillin-tazobactam, meropenem and ceftazidime [4].

Subdural empyema (SDE) may be a complication of sinusitis, meningitis, trauma, post surgical interventions, otitis media or secondary infection of a preexisting subdural hematoma [9]. Chronic calcified SDEs are extremely rare and they cannot be distinguished from chronic calcified subdural hematomas radiologically and pathologically [10]. MRI is superior to CT in demonstrating the characteristic imaging finding of a fluid collection surrounded by a contrast-enhancing rim [9]. Diffusion weighted imaging is sensitive to discriminate SDE from reactive subdural effusions and cystic neoplasms [9,11]. Reduced water diffusion is a characteristic feature of the purulent center of subdural inflammatory collections as well as in other pyogenic infections such as ventriculitis. Peripheral calcification occurs between 3 and 12 months after the onset, so the patients have a prior history of intracranial incident [10]. In the current case, the family of the patient denied any previous history of head injury, trauma, or infectious diseases, so the cause of this process is enigmatical.

To our knowledge there is only one report describing an intracranial infection caused by Achromobacter denitrificans presents as meningitis in the literature. This report is the first case which demonstrates an intracranial abscess formation due to this unique microorganism. Prompt identification of the pathogen and proper antibiotic treatment following bacterial isolation are fundamental in searching for unusual microorganisms when intracranial abscess occurs in patients with severe comorbidities.

## Competing Interests

The authors declare that they have no competing interests.
